# Mesenteric cystic lymphangioma: A case report

**DOI:** 10.1016/j.ijscr.2021.105659

**Published:** 2021-02-19

**Authors:** Ahmad K. Abdulraheem, Ahmed H. Al Sharie, Majd H. Al Shalakhti, Saleh Y. Alayoub, Hamzeh M. Al-Domaidat, Amin E. El-Qawasmeh

**Affiliations:** a Department of General Surgery and Urology, Faculty of Medicine, Jordan University of Science & Technology, Irbid, 22110, Jordan; b Faculty of Medicine, Jordan University of Science & Technology, Irbid, 22110, Jordan; c Inter, King Abdullah University Hospital, Jordan

**Keywords:** Mesenteric cystic lymphangioma, Case report, Intraabdominal lymphangioma, Surgical resection, Radiological findings

## Abstract

**Introduction and importance:**

Mesenteric cystic lymphangiomas are rare benign lesions of the abdominal cavity characterized by lymphatic vessels malformation with an unknown etiology. Despite the silent clinical course of mesenteric cystic lymphangiomas, they are considered as clinically tricky lesions with an immense spectrum of presentations.

**Case presentation:**

We present a case of abdominal mesenteric cystic lymphangioma in a 1-year 9-month-old female patient, who complained of fever and abdominal pain for 10 days duration. Laboratory investigations, abdominal X-ray, ultrasonography, computed tomography and histopathological examination were all used to establish the diagnosis.

**Clinical discussion:**

A trial of true-cut biopsies performed by an interventional radiologist was not informative, so a multidisciplinary team decision was made to excise the mass. Intraoperative findings include multiloculated fused cystic lesion (8.0 × 5.0 × 4.0 cm) on the descending mesocolon. Histopathological examination revealed the diagnosis of a mesenteric cystic lymphangioma. The postoperative period was not complicated.

**Conclusion:**

Mesenteric cystic lymphangiomas are mostly asymptomatic in nature, yet predisposed to life threating events. Surgical excision is the modality of treatment characterized by low recurrence rate and a non-complicated postoperative period.

## Highlights

• Mesenteric cystic lymphangiomas are rare benign lesions characterized by lymphatic vessels malformation with an unknown etiology.
• Although, mesenteric cystic lymphangiomas are rare but should be included in the differential diagnosis of abdominal pain.
• Surgical resection of mesenteric lymphangiomas should be performed as fast as possible to avoid many serios complications.

## 1 Introduction

Mesenteric cystic lymphangiomas (MCLs) are rare abdominal benign malformation of the lymphatic vessels with an incidence of 1 per 250,000 [1]. Representing 5–6% of pediatric benign tumors with a male predominance [2,3]. Around 65% of MCLs are discovered after birth and the rest will be eventually diagnosed by the age of 2 years [4]. The etiology of MCLs is not fully understood or studied, but several theories were proposed including the embryonal developmental theory, which is currently, the most relevant one since the majority of MCLs are firstly identified in the pediatrics age group. On the other hand, an acquired obstruction formed by hemorrhagic or an inflammatory process in the lymphatic vessels could lead to such cystic changes [5]. Clinically, MCLs have a wide range of presentations which varies from an asymptomatic incidentally discovered lesion to the case of acute abdomen. MCLs have been previously reported to mimic a variety of pathologies including appendicitis [6], pancreatitis [7] and even malignancies [8]. Patients mainly present with abdominal pain, abdominal distension or a palpable abdominal mass [9]. Ultrasonography, computed tomography and magnetic resonance imaging are all radiological techniques utilized in the evaluation of MCLs [3,10,11]. Usually, the majority of MCLs possess a silent clinical course but predisposed to bleeding, torsion and even rupture [12]. The gold standard treatment for MCLs is surgical resection [13].

## 2 Case presentation

A 1-year 9-month-old female patient with non-significant past medical or surgical history, a full-term product of a non-complicated vaginal delivery with no neonatal intensive care unit admissions was referred to our center for further investigations regarding the diagnosis of an intraabdominal mass versus an abdominal abscess. The patient complained of abdominal pain and fever for 10 days duration. The pain was located in the epigastric area radiating to left upper quadrant, and associated with nausea, vomiting and anorexia. The pain was severe enough to awaken the patient from her sleep. The fever was intermittent in nature with maximum recorded value of 39 °C orally, responded well to paracetamol. Upon physical examination, an epigastric tenderness was observed associated with left upper quadrant guarding to palpation. Otherwise, the abdomen was soft and lax. The suspected mass could not be palpated.

**Fig. 1:**
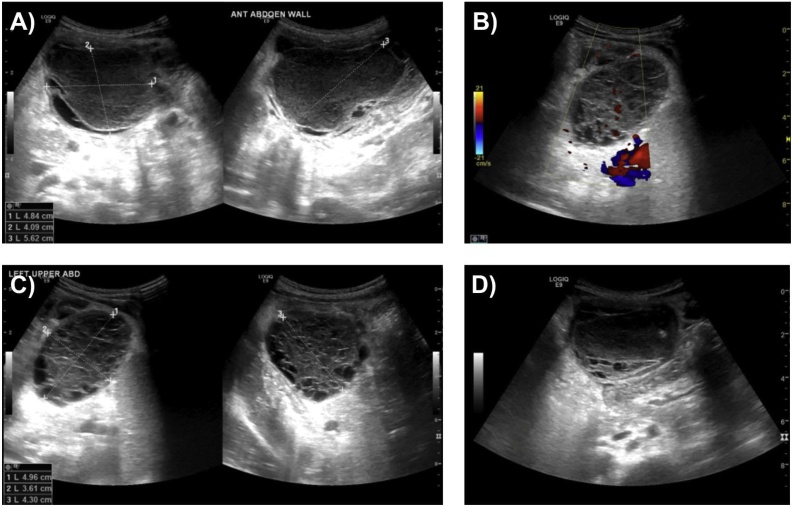
Abdominal ultrasonography (A–D) revealed a well-defined, multiloculated, cystic lesion seen in the epigastric region and extending into the left upper quadrant. The largest locule measured about 4.8 × 4.0 × 5.6 cm seen in the epigastric region containing swirling debris suggesting of an organized abscess secondary to infected mesenteric cyst or a lymphangioma.

**Fig. 2:**
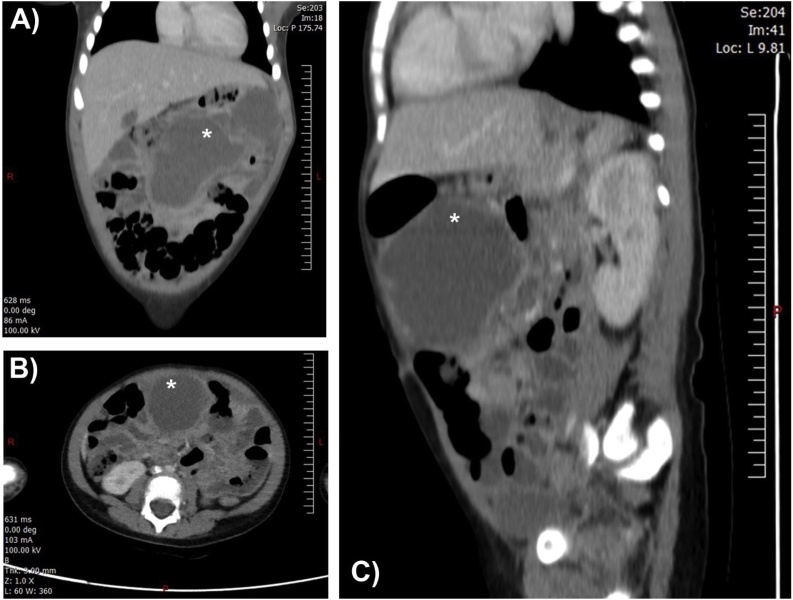
CT scan images at the coronal (A), axial (B), and sagittal planes (C), revealed a multiloculated thick enhancing-walled fluid collection seen in the mesentery anteriorly and extending to the left upper quadrant and flank region.

Laboratory investigations (Table 1), abdominal X-ray and ultrasonography were performed (Fig. 1). Ultrasonography revealed a well-defined, multiloculated, cystic lesion seen in the epigastric region and extending into the left upper quadrant with foci of calcification seen with posterior acoustic enhancement, with no hyperemia seen on Doppler images. The largest locule measured about 4.8 × 4.0 × 5.6 cm seen in the epigastric region containing swirling debris. Findings highly suggest an organized abscess secondary to infected mesenteric cyst or a lymphangioma. Liver, spleen and pancreas were all homogenous with no focal lesions. The long axis of the liver and spleen was 8.7 and 6.7 cm, respectively. Both kidneys appear normal in size, shape and echotexture with no hydronephrosis. The long axis of the right and left kidneys was 7.2 and 7.1 cm, respectively. Gallbladder had a smooth outline with no stones. No intra- or extra-hepatic biliary dilatation were seen. Subsequent computed tomography (CT) imaging with intravenous contrast (Fig. 2) revealed a multiloculated thick enhancing-walled fluid collection seen in the mesentery anteriorly and extending to the left upper quadrant and flank region. The largest locule is seen in the anterior abdomen measuring 6.7 × 6.2 cm containing two tiny foci of calcifications which caused a mass effect to adjacent small and large bowel loops displacing them posteriorly and laterally. The lesion was associated with sub-centimetric enhancing locoregional lymph nodes with the largest measuring about 0.5 cm in the short axis. A mild amount of fluid was seen in the pelvis. CT scan finding along with ultrasonography results suggest an organized abscess secondary to infected mesenteric cyst or a lymphangioma. A trial of true-cut biopsies performed by an interventional radiologist was not informative in which the histopathological examination of three needle core biopsies measuring 1.5, 1.5 and 0.5 cm indicated fragments of fibrinoid material with no viable cells and composed of skeletal muscles and fibrous tissue, precluding proper diagnosis.

**Table 1:** The diagnostic laboratory tests performed.

Test	Value	Test unit
**Complete blood count (CBC)**		
White cell count	21.8	× 10^3^ mm^3^
Red blood corpuscles count	3.89	× 10^6^ mm^3^
Hemoglobin (Hb)	8.70	g/dL
Hematocrit (HCT)	26.10	%
Mean cell volume (MCV)	67.00	μm^3^
Mean cell hemoglobin (MCH)	22.40	pg/cell
Mean cell hemoglobin concentration (MCHC)	33.50	g/dL
Red cell distribution width (RDW)	16.40	%
Platelet count	832.00	× 10^3^ mm^3^
Mean platelet volume	6.50	μm^3^
Neutrophils	71.70	%
Lymphocytes	20.00	%
Monocytes	6.40	%
Basophils	0.40	%
Eosinophils	1.50	%
**Liver function test (LFT)**		
Total protein	69.90	g/L
Albumin	38.00	g/L
Total bilirubin	1.90	μmol/L
Direct bilirubin	1.10	μmol/L
Alkaline phosphatase (ALP)	142.00	U/L
**Kidney function test (LFT)**		
Sodium (Na^+^)	138.00	μmol/L
Potassium (K^+^)	4.33	μmol/L
Urea	1.10	μmol/L
Creatinine	19.00	μmol/L
**Coagulation profile**		
Partial thromboplastin time (PTT)	32.40	Seconds
Prothrombin time (PT)	13.10	Seconds
PT-INR	0.97	
Fibrinogen	764.00	mg/dL
**Others**		
C-reactive protein (CRP)	203.05	mg/dL
Erythrocyte sedimentation rate (ESR)	83.00	mm/h
Lipase	10.00	U/L
Lactate dehydrogenase (LDH)	633.00	U/L
Carcinoembryonic antigen (CEA)	2.78	ng/mL
Human chorionic gonadotropin (β-HCG)	0.10	U/L
Blood culture	Negative	
Quantiferon (Latent tuberculosis test)	Negative	

**Blood smear results: RBCs**: appear microcytic normochromic with slight anisocytosis, slight polychromasia, few elliptocytes and target cells seen. **WBCs**: Neutrophil dominancy and showing toxic granulation. **Platelets:** Thrombocytosis with normal morphology.

**Fig. 3:**
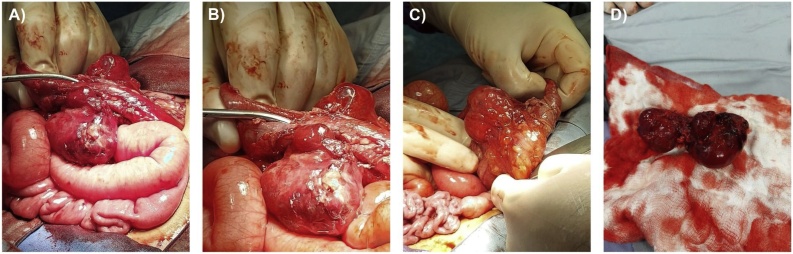
Intraoperative images (A-C) representing multiloculated fused cystic lesions on the descending mesocolon. The excised cystic mesenteric lymphangioma measured 8.0 × 5.0 × 4.0 cm (D).

**Fig. 4:**
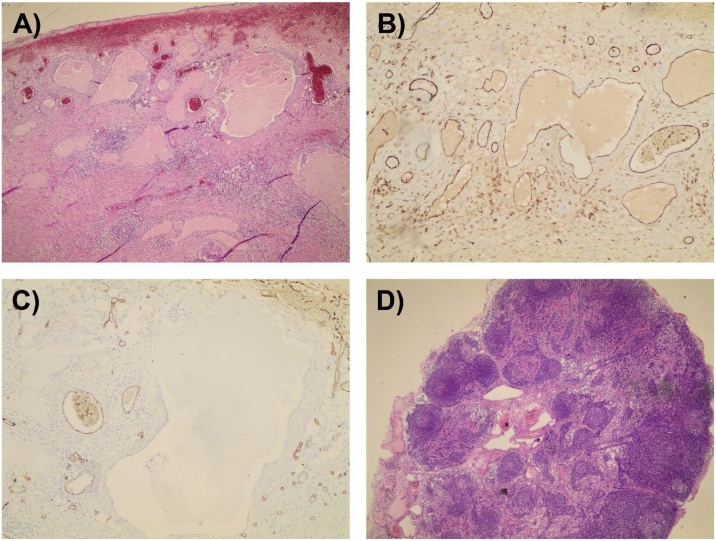
Histopathological examination of the excised MCL revealed a variably sized thin-walled vascular spaces almost devoid of blood and containing pale pink fluid mostly representing lymph (A). The spaces are lined by a layer of endothelial cells highlighted by CD31 (B) and CD34 (C) immunohistochemical staining. A representative sample reassembling one of the reactive excised lymph nodes (D).

A Multidisciplinary team decision was made to excise the mass by the pediatric surgery team. Intraoperative findings include (Fig. 3): multiloculated fused cystic lesions (8.0 × 5.0 × 4.0 cm) on the descending mesocolon. Descending colon loops were healthy and viable. Complete excision of the mass was performed with preservation of the attached descending colon through a midline laparotomy incision. Histopathological examination of the excised mass revealed a variably sized thin-walled vascular spaces almost devoid of blood and containing pale pink fluid mostly representing lymph. Such spaces are surrounded by loose fibrous tissue with inflammatory cellular infiltrate (mainly eosinophils and plasma cells) with lymphoid follicles (Fig. 4A). The spaces are lined by a layer of endothelial cells highlighted by CD31 and CD34 immunohistochemical staining (Fig. 4**B** and **C**). No evidence of malignancy was observed.

Postoperatively, a nasogastric (NG) tube was inserted, and the patient was on the following medication: vancomycin (165 mg, Q6h), piperacillin/tazobactam (1 g, Q6h), diclofenac suppositories (12.5 mg, Q12 h) and paracetamol (pro re nata). Medications were stopped at day 5 and the NG tube was removed at day 3 postoperatively. The patient was doing well, vitally stable, passed flatus on day 2, passed stool at day 4 and discharged after 5 days postoperatively. The patient was followed 1 week after the surgery in an outpatient clinic, the wound was healthy, not infected with normal bowel motion and no fever spikes, abdominal pain or abdominal distention.

## 3 Discussion

Mesenteric cystic lymphangiomas (MCLs) are clinically challenging abdominal lesions with a wide spectrum of clinical presentations ranges from an asymptomatic incidentally discovered finding to the case of acute abdomen. MCLs have been previously reported to mimic appendicitis with right lower quadrant pain associated with fever and leukocytosis [[14], [15], [16]]. In addition, a growing body of the literature has described the association between MCLs and intestinal volvulus [17,18], the patient in such setting will present with abdominal pain, abdominal distension, nausea and vomiting, an emergency surgical excision with partial bowel resection if needed should be performed [[19], [20], [21]]. Another misleading clinical presentation is malignancy mimicking setting in which the patient presents with weight loss, abdominal pain, postprandial fullness and loss of appetite as previously reported in a 27-year-old male patient diagnosed with an ileal MCL [8]. Other presentations include pancreatitis like [22], microcytic anemia and thrombocytosis [23] and gynecologic malignancy mimicking [24].

The differential diagnosis of MCL include mesenteric cysts, tuberculosis, tumor metastasis, hydatid disease, bowel adenocarcinomas, and other rare mesenteric malignancies [13]. The most common clinical complain of patients with MCLs is abdominal pain followed by abdominal fullness and palpable non-symptomatic mass. The median size of MCL after resection is 12.5 cm in which 50% of cases required bowel resection [9]. Although, MCLs have a moderately reported sizes, several studies described giant forms of MCLs [[25], [26], [27]], Bang et al. even described a giant 16 Kg MCL in a 46 -year-old female patient complained of progressive abdominal distension which was surgically excised [28]. Ultrasonography is considered as a sensitive and partially specific radiological tool for the assessment of MCLs which usually reveals a multicystic lesion with septations or sharply defined cystic mass, but previous report suggests that ultrasonography could mess such cystic masses [3,29]. Sonographic prenatal detection of MCLs is rare, however, Mostofian et al. reported a prenatal detection of a MCL in a 31-year-old female patient at the 25th week of gestation in which sonography showed two masses in the left and right sides characterized as multiseptated cystic deformable lesion with a solid mass [30]. CT scan remains the best radiological technique for evaluating MCLs for its abilities to determine the lesion’s size, its anatomical relations, density and enhancement properties, but magnetic resonance imaging (MRI) is more specific in determining the cyst contents [10,11]. Histopathological features of MCLs include thin-walled lymphatic vessels, attenuated endothelial lining, loose connective tissue, lymphoid aggregates and the presence of smooth muscles. Several immunohistochemical studies should be used for detailed histopathological examination including CD31, CD34, CD45 factor VIII-related antigen, HMB-45, D2-40 and calretinin [31,32].

The benign asymptomatic nature of MCLs are predisposed to bleeding, torsion and even rupture, leading to life-threatening emergencies which requires an early surgical excision to avoid such complications [1]. The vague clinical scenario exhibited by such lesions especially in emergencies endorse a lot of physicians to perform urgent surgical explorations. The vast majority of MCLs requires simple surgical excision which could be associated with partial bowel resection, MCLs exhibit a non-complicated postoperative period with low recurrence rate as previously described [33]. Many surgical procedures performed to excise MCLs are laparotomy techniques [34]. However, laparoscopic interventions have been reported [13,35,36]. This case has been reported in line with the SCARE guideline [37].

## 4 Conclusion

We yet report another case of MCL in a 1-year 9-mounth old female patient complained of fever and abdominal pain for 10 days duration. Although, mesenteric cystic lymphangiomas are rare but should be included in the differential diagnosis of abdominal pain especially in pediatric age group. Early surgical resection is highly endorsed even if it is asymptomatic to avoid many serious complications.

## Declaration of Competing Interest

The authors report no declarations of interest.

## Funding

This case report was not funded.

## Ethical approval

The presented case was reported according to the ongoing regulations of case reports and case series in the King Abdullah University Hospital (KAUH) as stated by the institutional review board committee. Case reports are exempt from ethical approvals in our center.

## Consent

Written informed consent was obtained from the patient’s parents for publication of this case report and accompanying images. A copy of the written consent is available for review by the Editor-in-Chief of this journal on request.

## Author contribution

**Acquisition of data**: Abdulraheem AK, Al Sharie AH, Shalakhti MH, Alayoub SY.

**Analysis and interpretation of data**: Abdulraheem AK, Al Sharie AH, Shalakhti MH, Alayoub SY, Al-Domaidat HM, El-Qawasmeh AE.

**Drafting of manuscript**: Abdulraheem AK, Al Sharie AH, Shalakhti MH, Alayoub SY, Al-Domaidat HM, El-Qawasmeh AE.

**Critical revision**: Abdulraheem AK, Al Sharie AH, Shalakhti MH, Alayoub SY, Al-Domaidat HM, El-Qawasmeh AE.

## Registration of research studies

Not applicable.

## Guarantor

Ahmad K. Abdulraheem and Saleh Y. Alayoub.

## Provenance and peer review

Not commissioned, externally peer reviewed.

## References

[1] Gunadi (2019). Pediatric patients with mesenteric cystic lymphangioma: a case series. Int. J. Surg. Case Rep..

[2] Katı Ö., Güngör Ş., Kandur Y. (2018). Mesenteric cystic lymphangioma: case report. J. Pediatric Surg. Case Rep..

[3] Konen O. (2002). Childhood abdominal cystic lymphangioma. Pediatr. Radiol..

[4] Geraci G. (2006). Mesenteric cyst lymphangioma; a case report and literature review. Ann. Ital. Chir..

[5] Losanoff J.E. (2003). Mesenteric cystic lymphangioma. J. Am. Coll. Surg..

[6] Francesco G. (2012). An unusual cause of “appendicular pain” in a young girl: mesenteric cystic lymphangioma. J. Surg. Case Rep..

[7] Akwei S., Bhardwaj N., Murphy P.D. (2009). Benign mesenteric lymphangioma presenting as acute pancreatitis: a case report. Cases J..

[8] Hureibi K., Sunidar O.A. (2014). Mesenteric cystic lymphangioma mimicking malignancy. BMJ Case Rep..

[9] Su C.M. (2007). Single-Centre results of treatment of retroperitoneal and mesenteric cystic lymphangiomas. Dig. Surg..

[10] Mabrut J.Y. (2002). Les lymphangiomes kystiques du mésentère et du méso-côlon. Prise en charge diagnostique et thérapeutique. Annales de Chirurgie.

[11] Thiam O. (2019). Cystic mesenteric lymphangioma: a case report. Int. J. Surg. Case Rep..

[12] Gunadi (2019). Pediatric patients with mesenteric cystic lymphangioma: a case series. Int. J. Surg. Case Rep..

[13] Nagano H. (2019). Cystic lymphangioma in the peripheral jejunal mesentery in an adult and excision with laparoscopic-assisted surgery: a case report. World J. Surg. Oncol..

[14] Kurbedin J. (2017). When fever, leukocytosis, and right lower quadrant pain is not appendicitis. Pediatr. Emerg. Care.

[15] Wake S., Abhyankar A., Hutton K. (2013). Abdominal cystic lymphangioma mimicking appendicitis. Eur. J. Pediatric Surg. Rep..

[16] Mesić M. (2013). Cystic lymphangioma of jejunal mesentery mimicking acute appendicitis: case report. Acta Clin. Croatica.

[17] de Vries J.J. (2007). Mesenterical lymphangiomatosis causing volvulus and intestinal obstruction. Lymphat. Res. Biol..

[18] Jang J.H. (2009). Small bowel volvulus induced by mesenteric lymphangioma in an adult: a case report. Korean J. Radiol..

[19] Elukoti H.N. (2015). Mesenteric lymphangioma presenting as ileal volvulus. J. Clin. Diagnostic Res..

[20] Poon M.C.-M. (2001). Mesenteric cystic lymphangioma presented with small bowel volvulus. Ann. Coll. Surg. Hong Kong.

[21] Losanoff J.E., Kjossev K.T. (2005). Mesenteric cystic lymphangioma: unusual cause of intra-abdominal catastrophe in an adult. Int. J. Clin. Pract..

[22] Akwei S., Bhardwaj N., Murphy P.D. (2009). Benign mesenteric lymphangioma presenting as acute pancreatitis: a case report. Cases J..

[23] Gambí Pisonero D. (2011). Microcytic anaemia and thrombocytosis as first signs of a mesenteric cystic lymphangioma. Cir. Esp..

[24] Maa J. (2009). Giant mesenteric cystic lymphangioma presenting with abdominal pain and masquerading as a gynecologic malignancy. Rare Tumors.

[25] Khattala K. (2011). Giant cystic lymphangioma of the small bowel mesentery: case report. Pan. Afr. Med. J..

[26] Rossini M. (2020). Symptomatic giant mesenteric cystic lymphangioma in adulthood. ACG Case Rep. J..

[27] Schest E. (2009). Giant mesenteric lymphangioma. Dig. Surg..

[28] Bang G.A. (2019). Giant sixteen kilogram lymphangioma mesenteric cyst: an unusual presentation of a rare benign tumour. Int. J. Surg. Case Rep..

[29] Chen J., Du L., Wang D.-R. (2018). Experience in the diagnosis and treatment of mesenteric lymphangioma in adults: a case report and review of literature. World J. Gastrointestinal Oncol..

[30] Mostofian E. (2004). Prenatal sonographic diagnosis of abdominal mesenteric lymphangioma. J. Ultrasound Med..

[31] Riahinezhad M. (2015). Two unusual sites of cystic lymphangioma in a child: a report of imaging profile with surgical and histopathologic findings. Adv. Biomed. Res..

[32] Hanganu E. (2017). A histopathological diagnosis of mesenteric cystic lymphangioma, clinically misdiagnosed as simple mesenteric cyst - case report. Rom. J. Morphol. Embryol..

[33] Egozi E.I., Ricketts R.R. (1997). Mesenteric and omental cysts in children. Am. Surg..

[34] Makni A. (2012). Surgical management of intra-abdominal cystic lymphangioma. Report of 20 cases. World J. Surg..

[35] Târcoveanu E. (2016). Laparoscopic treatment of intraabdominal cystic lymphangioma. Chirurgia (Bucur).

[36] Spolianski G. (2019). Laparoscopic exploration and treatment for a mesenteric cyst lymphangioma in adults. ANZ J. Surg..

[37] Agha R.A., Franchi T., Sohrabi C., Mathew G., for the SCARE Group (2020). The SCARE 2020 guideline: updating consensus surgical CAse REport (SCARE) guidelines. Int. J. Surg..

